# Self-Care Behaviors and Its Associated Factors in Adult Congenital Heart Disease: A Cross-Sectional Study

**DOI:** 10.3390/jcdd13060232

**Published:** 2026-05-28

**Authors:** Xiaotong Pan, Xinfang Zhang, Yonglin Wang, Xiaoxia Chen, Xi Cao

**Affiliations:** 1 Guangdong Provincial People’s Hospital (Guangdong Academy of Medical Sciences), Southern Medical University, Guangzhou 510080, China; panxt23@mail3.sysu.edu.cn (X.P.); zhangxinfang@gdph.org.cn (X.Z.); wangyonglin@gdph.org.cn (Y.W.);; 2 School of Nursing, Sun Yat-sen University, Guangzhou 510089, China

**Keywords:** adult congenital heart disease, self-care behaviors, associated factors, cross-sectional study

## Abstract

Aim: To investigate self-care behaviors and their associated factors in adult patients with congenital heart disease. Design: A cross-sectional study. Methods: This study was conducted among 225 adult patients with congenital heart disease. Data on demographic characteristics, self-care behaviors, knowledge of congenital heart disease, social support, and self-efficacy were collected. Multiple linear regression was used to explore factors associated with self-care behaviors. Result: The mean scores for self-care maintenance, self-care monitoring, and self-care management were 63.87 ± 9.19, 55.30 ± 13.64, and 83.99 ± 11.67, respectively. Using a cutoff score of 70 for adequate self-care, 27.1%, 15.1%, and 83.6% of participants achieved adequate levels in maintenance, monitoring, and management, respectively. Multivariable linear regression analysis revealed that classification of congenital heart disease complexity (B = 1.867, 95% CI [0.427–3.307], *p* = 0.011), self-efficacy (B = 0.187, 95% CI [0.105–0.270], *p* < 0.001), and social support utilization (B = 1.513, 95% CI [0.772–2.253], *p* < 0.001) were independently associated with self-care maintenance. Furthermore, knowledge questionnaire score (B = 0.158, 95% CI [0.034–0.281], *p* = 0.013) was independently associated with self-care monitoring. Objective support (B = 0.743, 95% CI [0.115–1.370], *p* = 0.021) and social support utilization (B = 0.178, 95% CI [0.035–2.121], *p* = 0.043) were independently associated with self-care management. Conclusions: Chinese adult congenital heart disease patients showed poor self-care maintenance and monitoring behaviors despite their adequate self-care management behaviors. Disease knowledge, social support, and self-efficacy may affect self-care behaviors in patients with congenital heart disease.

## 1. Background

Adult congenital heart disease (ACHD) refers to a population of patients with congenital cardiac defects who survive into adulthood, regardless of whether their defects have been corrected. According to recent epidemiological data, approximately 50 million adults are currently living with ACHD worldwide, and this number is projected to continue increasing [1]. In China, approximately 2.024 million individuals are living with congenital heart disease (CHD), with adults accounting for 53.48% of all cases; furthermore, adults constitute approximately 74.4% of hospitalized CHD patients [2,3]. Notably, around 90% of CHD patients now survive to adulthood [4], and survival rates extend beyond 60 years for 75% of those with moderate and 40% with severe complex lesions [5,6]. Consequently, the ACHD population is steadily expanding, presenting growing challenges for long-term disease management. However, the burden of long-term complications varies substantially by anatomical complexity. Patients with simple lesions predominantly face arrhythmias and infective endocarditis [7]; those with moderate-to-severe complexity are at higher risk for heart failure, pulmonary hypertension, cerebrovascular events, and multi-organ dysfunction (e.g., hepatic or renal impairment) [8,9,10]. Psychological disorders represent a ubiquitous concern across all complexity tiers during prolonged survival [11,12].

In the face of these complex health challenges, in addition to evidence-based treatment, self-care is crucial for ACHD patients. Self-care refers to the ability of patients, with the assistance of healthcare professionals and primary caregivers, to consistently and effectively implement a series of disease-related management behaviors over the long term [13]. In the situation of ACHD, self-care encompasses a range of activities including self-care maintenance behaviors (e.g., healthy diet, exercise, weight management, alcohol and tobacco restriction, and vaccination to maintain the stability of health, symptom monitoring behaviors and self-care management behavior, as well as preventive actions targeting specific complications like infective endocarditis and pregnancy-related issues [5]. A study has shown that ACHD patients exhibit low levels of self-care behaviors, particularly in symptom monitoring and management [14]. A large-scale multicenter study involving 15 countries demonstrated that the overall self-management behavior among ACHD patients was moderately low, with substantial variation observed across nations [15].

A comprehensive understanding of the underlying influencing factors is essential when developing self-care strategies for ACHD patients. Existing research has demonstrated that self-care behaviors in ACHD patients are shaped by the interplay of multiple factors, primarily encompassing sociodemographic characteristics, disease-related attributes, and psychosocial determinants. Regarding demographic profiles, age, sex, educational attainment, and employment status have all been significantly associated with self-care behaviors [15]. Self-efficacy, as a positive psychosocial construct, exerts beneficial effects on physical and psychological adaptation as well as health-promoting behaviors among ACHD patients [16]; however, findings regarding the strength of association between self-efficacy and self-care behaviors remain inconsistent across studies conducted domestically and internationally [14,17,18]. In terms of social support, ACHD patients universally express needs for social assistance and emotional support, the fulfillment of which directly influences patients’ social independence and adaptive capacity, thereby affecting self-care behaviors [19,20]. Nevertheless, the direct mechanisms through which social support impacts self-care behaviors in Chinese ACHD patients warrant further investigation. With respect to disease knowledge, previous studies have predominantly indicated a positive correlation between disease cognition and health behaviors among ACHD patients [21], yet counterintuitive findings have emerged suggesting that greater awareness of disease-related risks is associated with reduced physical activity levels, whereas general health knowledge demonstrates no such relationship [22], underscoring the complexity of the knowledge–behavior nexus.

Despite these conflicting findings, prior research has predominantly focused on Western populations, limiting the direct generalizability of existing evidence to Chinese patients. Furthermore, few studies have simultaneously examined the independent contributions of multidimensional factors (i.e., capability, opportunity, and motivation) to self-care behaviors within a unifying theoretical framework in ACHD populations. Therefore, this study aimed to examine self-care behaviors—including self-care maintenance, self-care monitoring, and self-care management—and their associated factors in Chinese ACHD patients. The COM-B model was employed as a conceptual framework to guide the selection of predictor domains: disease knowledge (representing capability), self-efficacy (representing motivation), and social support (representing opportunity) [23]. We hypothesized that these three factors would be independently associated with each self-care dimension. These findings are expected to inform the development of effective self-care interventions for this population.

## 2. Methods

### 2.1. Study Design

A cross-sectional study was adopted.

### 2.2. Setting and Participants

Participants were recruited from a cardiac center in Guangzhou, China from September 2023 to November 2024. This center, a nationally renowned cardiac institution in China known for cardiovascular excellence, attracts a diverse group of ACHD patients from across the country, including those with unique and severe conditions. The inclusion criteria were: ≥18 years old, with a diagnosis of CHD confirmed by their cardiologists, able to communicate in Cantonese or Mandarin, and able to provide written informed consent.

Patients were excluded if they were in the terminal stage of illness such as cancer or had an unstable health condition (e.g., acute heart failure) or with a history of psychiatric illness or cognitive impairment.

The sample size was estimated using the confidence interval method for one-sample means in PASS software (version 15.0; NCSS, LLC, Kaysville, UT, USA). Based on a previous study reporting a mean self-care management score of 59.2 ± 18.7 [14], the initial required sample size was calculated as 150 participants, assuming a 95% confidence level and a margin of error of 3. After adjusting for an anticipated non-response and missing data rate of 20%, the final minimum required sample size for this study was 187.

### 2.3. Data Collection and Analysis

The principal researcher screened patients who visited the outpatient clinic for eligibility according to the inclusion and exclusion criteria. The eligible patients were then invited to participate in this study after explaining the purpose and procedures of this study. Participants were asked to complete a set of questionnaires independently after obtaining the written informed consent. The principal researcher checked the questionnaires for completeness upon returning and asked participants to complete if there were missing data.

Continuous variables were presented as mean ± standard deviation (SD), and categorical variables were expressed as frequencies (percentages). Nonparametric rank-sum tests (Mann–Whitney U or Kruskal–Wallis H) were employed to examine the associations between different sociodemographic characteristics, disease-related factors, and self-care. Pearson correlation analysis was conducted to explore the relationships between self-care (self-care maintenance, self-care monitoring, and self-care management) and disease knowledge, social support, and self-efficacy. All variables with a *p*-value < 0.05 in the univariate analysis, together with age, sex, and education level, were entered into a multiple linear regression model to identify factors associated with self-care. To ensure that all predetermined candidate variables were included in the model, the enter method was used for multiple linear regression analysis. Variables with a variance inflation factor (VIF) > 10 were considered to have severe multicollinearity and were therefore excluded from the model; subsequently, the VIF values of all remaining variables were re-examined to ensure they fell within an acceptable range (VIF < 5). All statistical analyses were performed using SPSS 25.0 (IBM Corporation, Armonk, NY, USA), and a two-sided *p*-value < 0.05 was considered statistically significant.

### 2.4. Measures

#### 2.4.1. Sociodemographic and Clinical Characteristics

A standardized data collection form was used in this study. Sociodemographic data included age, sex, educational level, marital status, employment status, household income, medical payment method, financial burden of medical expenses, place of residence, and commuting time to healthcare facilities. Disease-related information included disease duration, number of surgeries, comorbidities, implanted medical devices, New York Heart Association (NYHA) functional class, and classification of congenital heart disease complexity. The classification of congenital heart disease complexity was operationalized according to the anatomical complexity criteria outlined in the 2020 European Society of Cardiology (ESC) Guidelines [6] for the Management of Adult Congenital Heart Disease. Patients were stratified into three complexity tiers (mild, moderate, and severe) based on anatomical diagnosis, hemodynamic burden, and repair or palliation status. Specifically, the mild tier included isolated small ventricular septal defects, mild pulmonary stenosis, and simple repaired lesions without residual lesions; the moderate tier included coarctation of the aorta, repaired tetralogy of Fallot, Ebstein anomaly, and transposition of the great arteries following arterial switch operation; and the severe tier included univentricular heart, Fontan circulation, pulmonary atresia, Eisenmenger syndrome, and cyanotic heart disease. This classification was independently determined by two cardiologists specializing in adult congenital heart disease (ACHD) based on anatomical diagnosis, hemodynamic status, and surgical history documented in the medical records; disagreements were resolved by consensus.

#### 2.4.2. Self-Care Behavior and Self-Efficacy

The Self-care Index for ACHD (SCI-ACHD), adapted from the Self-Care of Heart Failure Index by McCabe et al. from Emory University, assessed self-care behavior and self-efficacy [14]. This 32-item scale includes four subscales: self-care maintenance (16 items), self-care monitoring (6 items), self-care management (4 items, only for patients with symptoms in the past month), and self-care confidence (6 items). All the subscales are used independently, with self-care reflected by self-care maintenance, self-care monitoring, and self-care management behaviors. Each item is rated on a Likert 4-point scale. Each subscale is independently scored, the raw score (the sum of the items in the subscale) is then standardized to a total of 100, with a higher score indicating better self-care behavior or higher self-care confidence. A score below 70 indicates inadequate self-care. The 70-point cutoff was applied for descriptive reporting only (e.g., frequency of inadequate self-care), while continuous scores were used for all analytic models.

In this study, the SCI-ACHD demonstrated acceptable internal consistency, with Cronbach’s alpha coefficients ranging from 0.613 to 0.846 across subscales. While the lower bound (0.60) is modest, this is consistent with the original validation study by McCabe et al., which reported similar coefficients (0.60–0.83) in a comparable ACHD population [14]. The heterogeneity of self-care behaviors in ACHD—encompassing diverse anatomical lesions and treatment trajectories—may contribute to lower internal consistency compared to homogeneous heart failure cohorts. Given that the SCI-ACHD is currently the only validated instrument for self-care assessment in the ACHD population, its use is justified despite modest reliability.

#### 2.4.3. Knowledge About Congenital Heart Disease

The Chinese version of Leuven Knowledge Questionnaire for Congenital Heart Disease (LKQCHD) was used to assess participants’ knowledge about congenital heart disease [24]. It comprises 27 multiple-choice questions and 1 open-ended question, assessing the knowledge of disease treatment, complications, prevention, physical activity, sexuality, genetics, contraception, and pregnancy planning. Male patients were asked to complete the first 25 questions, while female patients answered all questions. The accuracy rate is calculated as: accuracy rate = (number of correct questions/number of total questions) × 100%. The scale showed good content validity [24].

#### 2.4.4. Social Support

The Social Support Rating Scale (SSRS), developed by Xiao Shuiyuan [25], was used to assess social support. It comprises three dimensions including objective support, subjective support, and support utilization. The scale consists of 10 items, with a total score ranging from 12 to 66. Higher scores indicate greater social support. In this study, the scale showed good reliability with a Cronbach’s α of 0.788.

### 2.5. Ethical Consideration

This study was approved by the Hospital Ethics Committee and registered on the Chinese Clinical Trial Registry. Written informed consent was obtained from all participants before data collection. All scales used in the study that were based on other researchers’ work had been authorized by the original authors. The study was conducted in accordance with ethical guidelines, ensuring the protection of participants’ rights and confidentiality.

## 3. Results

### 3.1. Participants’ Characteristics

This study included 225 adult congenital heart disease (ACHD) patients. Their sociodemographic and disease-related characteristics are outlined in Table 1.

The mean age of participants was 34.82 ± 12.79 years old, with a range from 18 to 74 years, and 61.3% were female. Patients with college constituted the largest proportion (30.2%), and 60.4% were cohabiting or married. A total of 57.8% reported a mild financial burden due to the treatment. In terms of cardiac function, 59.1% of the patients had Class II, and 48.4% had a mild severity of congenital heart disease. The detailed sociodemographic and disease-related characteristics are outlined in Table 1.

### 3.2. Self-Care Behavior Among Patients with ACHD

The average scores for self-care maintenance, self-care monitoring, and self-care management were 63.87 ± 9.19, 55.30 ± 13.64, and 83.99 ± 11.67, respectively. Using the cutoff point of 70 for judging adequacy of self-care, 27.1% of the participants reported adequate self-care maintenance behaviors, 15.1% for self-care monitoring behaviors and 83.6% for self-care management behaviors. The poorest-performing behavior in the self-care maintenance dimension was “Make a plan with your heart doctor before becoming pregnant?”, with a mean item score of merely 1.45. In the self-care monitoring dimension, the lowest-scoring item was “Experience side effects of medication?” (mean score: 1.64). Regarding the self-care management dimension, the item “Call your doctor or nurse for guidance?” achieved the lowest score, with a mean of 3.19.

### 3.3. Univariate Analysis of Participants’ Self-Care

Bivariate correlation analysis revealed that self-care maintenance was significantly correlated with self-efficacy (r = 0.287, *p* < 0.05), disease knowledge (r = 0.178, *p* < 0.05), objective support (r = 0.139, *p* < 0.05), perceived social support (r = 0.231, *p* < 0.05), and social support utilization (r = 0.349, *p* < 0.05). Self-care monitoring was correlated with self-efficacy (r = 0.172, *p* < 0.05) and disease knowledge (r = 0.236, *p* < 0.05). Self-care management was associated with objective support (r = 0.211, *p* < 0.05) and social support utilization (r = 0.227, *p* < 0.05).

Furthermore, significant associations were observed between self-care maintenance and several sociodemographic and clinical variables: age (Z/H = −0.833, *p* = 0.045), sex (Z/H = −2.066, *p* = 0.039), marital status (Z/H = 8.069, *p* = 0.018), comorbidities (Z/H = 7.716, *p* = 0.021), number of cardiac surgeries (Z/H = 0.160, *p* = 0.016), and the classification of congenital heart disease complexity (Z/H = 6.803, *p* = 0.033). Marital status (Z/H = 2.173, *p* = 0.037), per capita monthly household income (Z/H = 6.635, *p* = 0.036), place of residence (Z/H = −2.202, *p* = 0.028), and disease duration (Z/H = 9.835, *p* = 0.020) were associated with self-care monitoring. Per capita monthly household income (Z/H = 13.492, *p* = 0.001) was associated with self-care management (Supplementary Materials Table S4).

### 3.4. Multivariable Linear Regression Analysis of Participants’ Self-Care

Variables with statistical significance in the univariate analysis were entered into the multivariable linear regression model. As shown in Table 2, classification of congenital heart disease complexity (B = 1.867, 95% CI [0.427–3.307], *p* = 0.011), self-efficacy (B = 0.187, 95% CI [0.105–0.270], *p* < 0.001), and social support utilization (B = 1.513, 95% CI [0.772–2.253], *p* < 0.001) were independently associated with self-care maintenance. Furthermore, knowledge questionnaire score (B = 0.158, 95% CI [0.034- 0.281], *p* = 0.013) was independently associated with self-care monitoring. Objective support (B = 0.743, 95% CI [0.115–1.370], *p* = 0.021) and social support utilization (B = 0.178, 95% CI [0.035–2.121], *p* = 0.043) were independently associated with self-care management.

## 4. Discussion

This study reveals a striking paradox in self-care behaviors among Chinese adults with congenital heart disease (ACHD). Despite suboptimal performance in self-care maintenance and monitoring, the cohort demonstrated significantly higher self-care management scores (83.99 ± 11.67) compared to those reported by McCabe et al. (59.2 ± 18.7) [14]. This pattern suggests that Chinese ACHD patients possess robust reactive capacities for acute exacerbations yet demonstrate insufficient engagement in proactive daily self-care routines. Several potential explanations for this disparity warrant consideration, though they remain speculative given the cross-sectional design and require empirical validation. China’s hospital-centric healthcare system—coupled with limited accessibility to ACHD-specific primary care—may inadvertently reinforce reactive rather than proactive health behaviors [26,27,28]. Additionally, China’s collectivist cultural context could position family members as active caregivers during acute illness episodes, wherein shared cognition and collaborative decision-making potentially augment patients’ management capabilities [29,30,31]. Consequently, elevated management scores may reflect dyadic- or family-level competencies rather than purely individual disease management skills. These interpretations are post hoc and should be examined in future comparative or qualitative studies. The alarming contrast between the 83.6% adequate management rate and the 15.1% monitoring rate raises critical concerns that patients may be managing symptoms that escaped detection through proper monitoring, potentially resulting in delayed treatment [32].

Regarding specific behavioral domains, the self-care maintenance subscale revealed that the item “making a plan with your cardiologist before becoming pregnant” received the lowest scores, indicating suboptimal reproductive health planning among Chinese ACHD patients. Female participants scored significantly higher on this item than male participants; nevertheless, a considerable proportion of women still failed to achieve adequate scores, suggesting that awareness of preconception planning remains insufficient even among those with greater perceived relevance. These findings align with previous research documenting that the majority of female ACHD patients fail to receive counseling regarding safe contraception and pregnancy [33,34,35,36,37]. By contrast, male patients demonstrated markedly lower scores, which likely reflects a critical gap in recognition that male ACHD patients also bear direct reproductive health responsibilities, including genetic risk assessment and counseling related to partner pregnancy planning, given the heritability of congenital heart defects. Burström’s study further demonstrated limited knowledge of reproductive health and risks during the adolescent-to-adult transition [38]. Collectively, these findings underscore the urgent necessity of integrating comprehensive reproductive health discussions into congenital heart disease care.

Similarly, within the self-care monitoring domain, the lowest-scoring item concerned “monitoring for medication side effects,” reflecting substantial difficulties in identifying and responding to adverse drug reactions. Akiyama reported that merely 39.4% of ACHD patients could name their medications and associated side effects [39], while another study indicated that 35.9% exhibited medication non-adherence coupled with inability to recognize adverse reactions [40]. Compounding these concerns, although ACHD patients demonstrated generally favorable performance in self-care management, the item “contacting healthcare providers for guidance” received the lowest scores, indicating underutilization of available resources during symptomatic episodes. This pattern mirrors the nursing challenges and unmet individual needs previously identified among ACHD populations [41].

To examine the factors underlying these behavioral patterns, we utilized the COM-B model as a theoretical framework to guide variable selection. Multivariable regression analyses revealed that the three self-care dimensions were influenced by distinct factors: self-care maintenance was independently associated with motivation (self-efficacy), opportunity (social support utilization), disease severity, marital status, and surgical history; self-care monitoring was associated with capability (disease knowledge); and self-care management was associated with opportunity (social support utilization) and objective support.

Delving deeper into self-care maintenance, self-efficacy was significantly associated with this domain, suggesting that enhancing patient confidence represents a potential strategy for improving daily adherence behaviors. Notably, this association diverged from previous findings [14,42], as self-efficacy was not significantly associated with monitoring or management in the present sample. This divergence may reflect differential task demands: maintenance behaviors consist of daily repetitive tasks that rely heavily on self-confidence to overcome implementation barriers, whereas monitoring requires sustained cognitive engagement, and management may be facilitated by external support systems—both potentially attenuating the independent role of individual self-efficacy [43]. This hypothesis requires validation in future research. Furthermore, anatomical complexity classification was positively associated with maintenance behaviors, which may indicate that patients with more complex lesions accumulate greater illness experience through repeated healthcare encounters, thereby reinforcing behavioral routines [44,45]. Crucially, social support utilization was also independently associated with maintenance, consistent with previous demonstrations that multidimensional social support promotes self-care maintenance in chronic heart disease populations [46,47]. These findings suggest that strengthening family–hospital–community integrated networks to translate implicit support into structured, accessible maintenance assistance may represent a promising intervention target [48]. Future intervention research could explore collaborative goal-setting between patients and providers to enhance behavioral change confidence [16,49], alongside strategies to increase patient engagement in health-related decision-making [50,51,52,53].

In contrast, self-care monitoring was predominantly influenced by knowledge levels, consistent with Fernandes et al. and Moons et al.’s findings [54]. Thus, improving disease knowledge represents a crucial measure for enhancing monitoring capabilities. Chiang et al. [55] and Cornett et al. [19] demonstrated that ACHD patients, particularly during the adolescent-to-adult transition, exhibit increased needs for disease-related knowledge and desire adaptive monitoring strategies to overcome setbacks and anxiety. Consequently, providing lifelong self-care education across all age groups, with particular emphasis on transition planning from adolescence, constitutes an essential clinical priority [56,57,58,59,60,61].

In self-care management, both social support utilization and objective support emerged as significant correlates, consistent with prior literature documenting the social needs and challenges inherent in ACHD management [62,63,64,65,66]. The influence of objective support—reflecting tangible, instrumental assistance—highlights the critical role of external support systems. This finding suggests that family and social networks may transform individual management behaviors into collaborative responses, thereby compensating for gaps in personal capability [67]. Accordingly, clinical interventions should focus on enhancing structural pathways for support utilization, such as establishing peer support networks, providing empowerment training for family caregivers, and leveraging community-based resources to translate available social support into explicit, accessible management assistance [48,68,69].

This study has several limitations. First, the cross-sectional design precludes causal inference, and residual confounding from unmeasured variables cannot be excluded. Second, convenience sampling may have introduced selection bias, limiting the generalizability of the findings. Third, the SCI-ACHD demonstrated modest internal consistency. Fourth, the regression models explained limited variance, suggesting the influence of additional unrecognized factors. Given these methodological constraints, the present findings are strictly hypothesis-generating and should not be interpreted as confirmatory. Future longitudinal studies with larger samples, using structural equation modeling or mediation analysis, are warranted to formally test the preliminary COM-B associations identified in this exploratory work and to clarify causal pathways. Only after such confirmatory evidence is available can theory-based intervention studies be rationally developed to inform self-care strategies for this population.

## 5. Conclusions

This study revealed that the population reported adequate self-care management behavior but inadequate monitoring and self-care maintenance behaviors. Self-efficacy, social support, and disease knowledge may affect the self-care behavior of this population.

## Figures and Tables

**Table 1:** Sociodemographic and disease characteristics of adult congenital heart disease patients (N = 225).

Variable	N (%)
Gender	
Male	87 (38.7)
Female	138 (61.3)
Age (years)	
18–44	176 (78.2)
>44	49 (21.8)
Education Level	
Below High School	59 (26.2)
High School	56 (24.9)
College	68 (30.2)
Bachelor’s or Above	42 (18.7)
Marital Status	
Single/Divorced/Widowed/Separated	89 (39.6)
Cohabiting/Married	136 (60.4)
Employment Status	
Employed	136 (60.4)
Unemployed/Retired	89 (39.6)
Household Income per Month (yuan)	
≤5000	106 (47.1)
5001–10,000	80 (35.6)
>10,000	39 (17.3)
Financial Burden relating to the Treatment and Healthcare	
No burden	39 (17.3)
Mild burden	130 (57.8)
Significant burden	45 (20.0)
Overwhelming burden	11 (4.9)
Place of Residence	
Rural	95 (42.2)
Urban	130 (57.8)
Travel Time to Specialized Care (hours)	
<1	50 (22.2)
1–4	136 (60.4)
>4	39 (17.4)
Variable	N (%)
Disease Duration (years)	
<1	24 (10.7)
1–5	85 (37.8)
5–10	27 (12.0)
>10	89 (39.6)
Number of Cardiac Surgeries	
0	143 (63.6)
1	64 (28.4)
2	13 (5.8)
≥3	5 (2.2)
Implanted Cardiac Devices	
Yes	8 (3.6)
No	217 (96.4)
NYHA functional class	
I	68 (30.2)
II	133 (59.1)
III	23 (10.2)
IV	1 (0.4)
Comorbidities	
0	133 (59.1)
1	73 (32.4)
2	14 (6.2)
≥3	5 (2.2)
The classification of congenital heart disease complexity	
Mild	109 (48.4)
Moderate	49 (21.8)
Severe	67 (29.8)

Note: Comorbidities include coronary atherosclerosis, hypertension, hyperthyroidism, tuberculosis, depression, pulmonary embolism, chronic obstructive pulmonary disease, thalassemia, and others. Implanted cardiac devices include permanent pacemakers, implantable cardioverter defibrillators, pulmonary artery stents, and others. The classification of severity of congenital heart disease refers to the diagnostic and classification standards proposed by the European Society of Cardiology in 2020.

**Table 2:** Multiple linear regression analysis of self-management behavior in ACHD patients.

Variable	B (95%CI)	SE	t	*p*	β	VIF
**Self-care Maintenance (N = 225)**						
Age	0.027 (−0.082–0.136)	0.055	0.494	0.622	0.038	1.772
Gender	2.160 (−0.114–4.435)	1.154	1.872	0.063	0.115	1.128
Education level	0.160 (−1.016–1.336)	0.597	0.268	0.789	0.019	1.450
Marital status	1.334 (−1.175–3.843)	1.273	1.048	0.296	0.079	1.714
Comorbidities	1.263 (−0.496–3.022)	0.892	1.415	0.158	0.098	1.444
Number of heart surgeries	1.439 (−0.116–2.995)	0.789	1.824	0.070	0.111	1.108
Classification of congenital heart disease complexity	1.867 (0.427–3.307)	0.730	2.556	0.011	0.176	1.425
Knowledge questionnaire score	0.064 (−0.011–0.138)	0.038	1.692	0.092	0.108	1.214
Self-efficacy	0.187 (0.105–0.270)	0.042	4.466	<0.001	0.290	1.265
Objective support	−0.244 (−0.718–0.230)	0.240	−1.015	0.311	−0.070	1.428
Subjective support	0.225 (−0.052–0.502)	0.141	1.598	0.111	0.121	1.722
Social support utilization	1.513 (0.772–2.253)	0.376	4.027	<0.001	0.262	1.274
**Self-care Monitoring (N = 225)**						
Age	0.108 (−0.071–0.286)	0.090	1.191	0.235	0.101	1.704
Gender	1.472 (−2.214–5.159)	1.870	0.787	0.432	0.053	1.063
Education level	−1.130 (−3.217–0.957)	1.059	−1.067	0.287	−0.089	1.637
Marital status	−1.468 (−5.393–2.457)	1.991	−0.737	0.462	−0.059	1.503
Household income per month	2.471 (−0.202–5.143)	1.356	1.822	0.070	0.135	1.309
Place of residence	1.986 (−2.054–6.026)	2.050	0.969	0.334	0.072	1.313
Disease duration	1.169 (−0.475–2.812)	0.834	1.402	0.162	0.093	1.034
Knowledge questionnaire score	0.158 (0.034–0.281)	0.063	2.516	0.013	0.179	1.204
Self-efficacy	0.114 (−0.017–0.244)	0.066	1.711	0.088	0.119	1.138
**Self-care Management (N = 214)**						
Age	−0.039 (−0.172–0.094)	0.067	−0.574	0.566	−0.043	1.281
Gender	3.329 (0.130–6.527)	1.622	2.052	0.041	0.140	1.061
Education level	−0.131 (−1.854–1.593)	0.874	−0.149	0.881	−0.012	1.453
Household income per month	1.335 (−0.933–3.603)	1.151	1.160	0.247	0.084	1.210
Objective support	0.743 (0.115–1.370)	0.318	2.334	0.021	0.170	1.215
Social support utilization	1.078 (0.035–2.121)	0.529	2.037	0.043	0.147	1.200

Self-care Maintenance: R^2^ = 0.293, F = 7.335, *p* < 0.001. Self-care Monitoring: R^2^ = 0.081, F = 2.375, *p* = 0.018. Self-care Management: R^2^ = 0.107, F = 3.511, *p* < 0.001.

## Data Availability

Data are available on request from the authors.

## Supplementary Materials

The following supporting information can be downloaded at: https://www.mdpi.com/article/10.3390/jcdd13060232/s1, Supplementary Table S1: Correlation between self-management behaviors and Knowledge questionnaire score in ACHD; Supplementary Table S2: Correlation between Self-management Behaviors and Social Support in Patients with ACHD; Supplementary Table S3: Correlation between self-management behaviors and self-efficacy in patients with ACHD; Supplementary Table S4: Univariate Analysis of Self-Management Behavior in Patients with ACHD.
