# Supplementary material for: Self-Care Behaviors and Its Associated Factors in Adult Congenital Heart Disease: A Cross-Sectional Study
Source: J Cardiovasc Dev Dis. 2026 May 28;13(6):232. doi: 10.3390/jcdd13060232 (PMC13300665; doi:10.3390/jcdd13060232)
Supplement: Supplementary file 1 [file jcdd-13-00232-s001.zip › jcdd-4289364-supplementary.pdf]

Supplementary Table S1 : Correlation between self-management behaviors and Knowledge questionnaire score in ACHD（N=225）

Variable	Knowledge questionnaire score
**Self-care Maintenance**	0.178**
**Self-care Monitoring**	0.236**
**Self-care Management**	0.051

Supplementary Table S2: Correlation between Self-management Behaviors and Social Support in Patients with ACHD（N=225）

Variable	Objective support	Subjective Support	Social support utilization
**Self-care Maintenance**	0.139*	0.231**	0.349**
**Self-care Monitoring**	0.095	0.090	0.018
**Self-care Management**	0.211**	0.019	0.227**

Supplementary Table S3 : Correlation between self-management behaviors and self-efficacy in patients with ACHD（N=225）

Variable	self-efficacy
**Self-care Maintenance**	0.287**
**Self-care Monitoring**	0.172**
**Self-care Management**	0.028

Supplementary Table S4 : Univariate Analysis of Self-Management Behavior in Patients with ACHD（N=225）

Variable	Self-care Maintenance			Self-care Monitoring			Self-care Management		
	$\bar{\chi}\pm S$	*Z/H*	*P*	$\bar{\chi}\pm S$	*Z/H*	*P*	$\bar{\chi}\pm S$	*Z/H*	*P*
**Age (years)**		-0.833	0.045		-0.809	-0.419		-0.073	0.942
18-44	63.56±9.16			55.21±12.78			83.93±12.05		
>44	64.99±9.28			55.61±16.50			84.21±10.31		
**Gender**		-2.066	0.039		-0.168	0.866		-1.775	0.076
Male	62.45±9.75			54.83±12.73			82.14±12.00		
Female	64.76±8.72			55.59±14.21			85.19±11.13		
**Education Level**		3.409	0.333		3.402	0.332		0.482	0.923
Below High School	64.19±8.63			54.38±13.76			83.18±11.52		
High School	63.14±9.27			54.84±14.71			84.39±10.40		
College	63.01±9.66			56.99±13.72			84.11±12.40		
Bachelor’s or Above	65.77±9.04			54.46±11.98			84.43±12.73		
**Marital Status**		8.069	0.018		2.173	0.037		3.640	0.162
Single/Divorced/Widowed/Separated	61.80±9.27			54.31±13.74			85.20±12.16		
Cohabiting/Married	65.22±8.90			55.94±13.59			83.20±11.32		

Variable	Self-care Maintenance			Self-care Monitoring			Self-care Management		
	$\bar{\chi}\pm S$	*Z/H*	*P*	$\bar{\chi}\pm S$	*Z/H*	*P*	$\bar{\chi}\pm S$	*Z/H*	*P*
**Employment Status**		-0.116	0.907		-0.466	0.642		0.171	0.679
Employed	63.95±9.29			55.70±13.67			83.92±11.37		
Unemployed/Retired	63.75±9.08			54.68±13.64			84.11±12.17		
**Household Income per Month**		2.766	0.251		6.635	0.036		13.492	0.001
≤5000	63.05±8.82			53.34±13.56			81.44±12.01		
5001-10000	63.98±9.15			56.35±13.41			87.71±9.75		
>10000	65.87±10.11			58.44±13.84			83.10±12.70		
**Payment Method for Medical Expenses**		1.180	0.554		0.544	0.762		3.056	0.217
Out-of-pocket	65.80±6.92			56.02±16.15			89.81±10.02		
Public expense	66.56±9.91			56.67±11.26			88.33±9.50		
Medical insurance	63.72±9.27			55.23±13.64			83.63±11.74		
**Financial Burden of Routine Treatment and Healthcare**		0.718	0.869		3.996	0.262		2.684	0.443
No burden	64.86±9.62			58.98±14.67			86.34±11.81		
Mild burden	63.46±8.95			54.49±13.14			83.87±11.96		

Variable	Self-care Maintenance			Self-care Monitoring			Self-care Management		
	$\bar{\chi}\pm S$	*Z/H*	*P*	$\bar{\chi}\pm S$	*Z/H*	*P*	$\bar{\chi}\pm S$	*Z/H*	*P*
Significant burden	64.31±9.50			53.61±13.04			82.95±10.78		
Overwhelming burden	63.35±9.99			58.71±16.92			81.67±11.65		
**Place of Residence**		-1.220	0.222		-2.202	0.028		-0.174	0.861
Rural	63.43±8.13			53.29±13.26			82.89±11.35		
Urban	64.18±9.90			56.76±13.78			84.85±11.88		
**Travel Time to Specialized Care (hours)**		1.331	0.514		2.285	0.319		1.111	0.574
<1	63.34±9.89			57.25±13.75			85.33±11.81		
1-4	63.81±8.99			54.93±13.16			83.59±11.27		
>4	64.74±9.07			54.06±15.19			83.78±13.02		
**Disease Duration (years)**		1.951	0.583		9.835	0.020		2.128	0.546
<1	66.34±10.26			47.57±14.32			82.58±12.04		
1-5	63.05±8.48			56.32±14.47			84.45±12.27		
5-10	63.77±8.72			57.25±10.94			87.00±6.84		
>10	64.01±9.68			55.81±13.64			83.04±12.09		

Variable	Self-care Maintenance			Self-care Monitoring			Self-care Management		
	$\bar{\chi}\pm S$	*Z/H*	*P*	$\bar{\chi}\pm S$	*Z/H*	*P*	$\bar{\chi}\pm S$	*Z/H*	*P*
**Number of Cardiac Surgeries**		0.160	0.016		3.410	0.333		5.231	0.156
0	62.81±9.10			56.03±12.70			83.95±11.72		
1	65.09±9.02			54.62±16.01			84.14±11.26		
2	67.79±8.90			53.85±10.93			88.19±10.93		
⩾3	68.44±11.18			46.67±12.98			73.33±13.69		
**Comorbidities**		7.716	0.021		0.983	0.612		1.540	0.463
0	62.96±9.40			55.42±13.20			84.68±11.21		
1	64.23±8.54			54.45±14.41			83.10±12.27		
2	71.09±8.49			58.63±15.37			84.62±13.54		
⩾3	62.50±6.72			55.00±10.79			78.33±9.50		
**Implanted Cardiac Devices**		-.0640	0.522		-0.236	0.813		-0.864	0.387
Yes	65.63±8.18			53.65±12.08			78.57±17.91		
No	63.80±9.23			55.36±13.72			84.18±11.42		
**NYHA functional class**		2.220	0.528		5.297	0.151		6.255	0.100

**Variable**	**Self-care Maintenance**			**Self-care Monitoring**			**Self-care Management**		
	$\bar{\chi}$±*S*	*Z/H*	*P*	$\bar{\chi}$±*S*	*Z/H*	*P*	$\bar{\chi}$±*S*	*Z/H*	*P*
Ⅰ	63.25±8.87			55.45±14.07			86.44±11.67		
Ⅱ	63.82±9.47			54.26±13.24			82.44±11.98		
Ⅲ	66.10±8.62			60.14±14.09			86.59±8.61		
Ⅳ	60.94			70.83	.		83.33		
**The classification of congenital heart disease complexity**		6.803	0.033		1.962	0.375		0.759	0.684
Mild	62.64±9.76			54.78±12.82			84.56±11.45		
Moderate	64.44±9.00			58.25±14.87			84.07±12.54		
Severe	65.43±8.13			53.98±13.90					

Note: Self-care management was included in only 214 individuals.
